# Intracellular/surface moonlighting proteins that aid in the attachment of gut microbiota to the host

**DOI:** 10.3934/microbiol.2019.1.77

**Published:** 2019-03-11

**Authors:** Constance J. Jeffery

**Affiliations:** Department of Biological Sciences, University of Illinois at Chicago, Chicago, IL 60607, USA

**Keywords:** moonlighting proteins, cell surface receptor, adhesion, microbiota, multifunctional proteins, protein function

## Abstract

The gut microbiota use proteins on their surface to form and maintain interactions with host cells and tissues. In recent years, many of these cell surface proteins have been found to be identical to intracellular enzymes and chaperones. When displayed on the cell surface these moonlighting proteins help the microbe attach to the host by interacting with receptors on the surface of host cells, components of the extracellular matrix, and mucin in the mucosal lining of the digestive tract. Binding of these proteins to the soluble host protein plasminogen promotes the conversion of plasminogen to an active protease, plasmin, which activates other host proteins that aid in infection and virulence. In this mini-review, we discuss intracellular/surface moonlighting proteins of pathogenic and probiotic bacteria and eukaryotic gut microbiota.

## 1. Introduction

**Figure 1.**
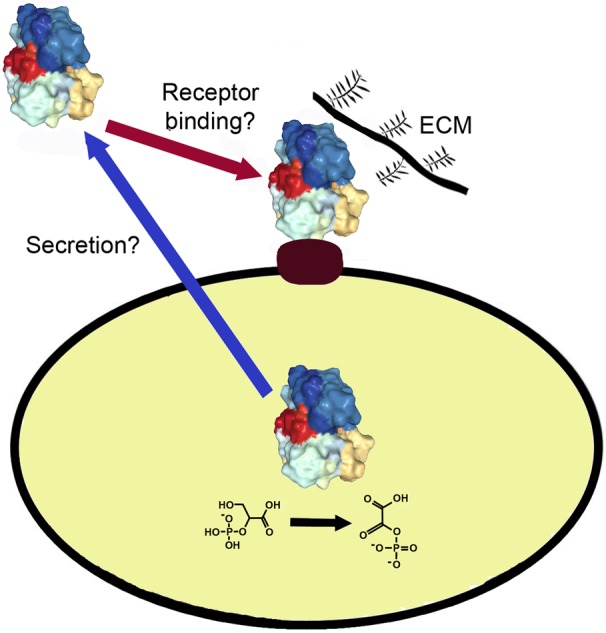
Intracellular proteins of the gut microbiota can be displayed on the cell surface and mediate interaction with the host. An enzyme or chaperone inside of the cell (protein structure) can also be present on the cell surface. In some cases, the protein enables the cell to bind to host proteins such as fibronectin, collagen and laminin in the extracellular matrix (ECM) or act as an adhesion to host cells. Interactions with the soluble host protein plasminogen can help it become converted to the active protease plasmin, which helps break down host tissues during infection. It is not known how most of the intracellular/cell surface proteins are secreted (blue arrow) or bind to the cell surface (red arrow).

Interaction of the gut microbiota with the host requires proteins that are on the cell surface to form and maintain interactions with host cells and tissues. These interactions are important for infection by pathogens and for the symbiotic relationships between the normal gut microbiota and the host. In recent years, many of these surface proteins have been found to be identical to cytoplasmic enzymes and chaperones. They belong to a larger group of proteins with multiple functions called moonlighting proteins. Moonlighting proteins are multifunctional proteins in which a single polypeptide chain performs two or more physiologically relevant biochemical or biophysical functions [1]. Hundreds have been identified and are described further in the online MoonProt Database (moonlightingproteins.org) [2]. Over 100 are cytoplasmic proteins that have a second function on the cell surface, often as adhesins that bind to host cells and tissues [3],[4] (Figure 1). This mini-review focuses on intracellular proteins that moonlight on the cell surface of bacterial and eukaryotic gut microbiota.

## 2. Examples

Dozens of intracellular proteins have been found on the surface of gut microbiota where they interact with host cell surface receptors, ECM, plasminogen or other proteins. These proteins are found to be moonlighting in many types of gut microbiota - pathogens and commensal bacteria, including both Gram negative and Gram positive species, yeast, and an amoeba.

### 2.1. Bacteria

#### 2.1.1. Pathogenic bacteria

One of the most commonly found intracellular/surface moonlighting proteins in pathogenic bacteria is Hsp60, a chaperone involved in protein folding. In the gut microbiome, it is also used on the cell surface as an adhesin in several species that cause diarrhea and other, sometimes serious or even lethal, complications. [5]–[8] (Table 1). The Gram positive spore forming *Clostridium difficile* (CDI or C-dif), causes tens of thousands of deaths in the US each year. *Helicobacter pylori* is a Gram negative bacterium that can be a normal part of the stomach biome but can also lead to chronic gastritis, ulcers and stomach cancer. The Gram negative *Salmonella enterica* serotype Typhimurium is an intracellular pathogen that causes food poisoning, salmonellosis, which is often spread by eating contaminated meat, eggs, or milk. *Listeria monocytogenes* is transmitted in contaminated unpasteurized dairy products and the reason pregnant women are recommended not to eat soft cheeses, such as brie, because it can cause meningitis in newborns. In addition to Hsp60 [9], Listeria uses another intracellular protein, alcohol acetaldehyde dehydrogenase [10], as an adhesin and Ami autolysin, a cell surface enzyme that cleaves cell wall glycopeptides, in a second role as an adhesin to mammalian cells [11].

**Table 1.** Moonlighting Proteins on the Surface of Gut Microbiota that interact with the Host.

	Cytoplasmic Function	Cell Surface Function	Reference
*Bifidobacteria*			
Bile salt hydrolase	hydrolase	binds plasminogen	20
DnaK	chaperone	binds plasminogen	20
Enolase	hydratase	binds plasminogen	19,20
Glutamine synthetase	synthetase	binds plasminogen	20
Phosphoglycerate mutase	mutase	binds plasminogen	20
			
*Lactobacillus johnsonii*			
Ef-Tu	elongation factor	Binds human cells and mucins	17
Hsp60	chaperone	binds mucins and epithelial cells	16
*Lactobacillus crispatus*			
Enolase	hydratase	binds plasminogen and laminin	13
Glucose 6-phosphate isomerase	isomerase	binds laminin, collagen	15
Glutamine synthetase	synthetase	binds plasminogen, fibronectin, laminin, collagen	15
*Lactobacillus plantarum*			
Enolase	hydratase	Binds fibronectin	12
GAPDH	dehydrogenase	binds mucin and Caco2 cells	18
*Lactobacillus acidophilus*			
GAPDH	dehydrogenase	binds mucin	14
*Clostridium difficile*			
Hsp60	chaperone	adhesin	5
			
*Helicobacter pylori*			
Hsp60	chaperone	adhesin	6,8
*Listeria monocytogenes*			
Alcohol acetaldehyde	dehydrogenase	adhesin	10
Hsp60 chaperone	chaperone	adhesin	9
Ami autolysin	autolysin	adhesin	11
*Salmonella enterica serotype Typhimurium*			
Hsp60	chaperonin	adhesin	7
*Candida albicans*			
Alcohol dehydrogenase (ADH1)	dehydrogenase	binds plasminogen	21
Enolase	hydratase	binds plasminogen	23
Fructose bisphosphate aldolase	aldolase	binds plasminogen	21
GAPDH	dehydrogenase	binds plasminogen, fibronectin, laminin	21,22
Peroxisomal catalase (CTA1)	catalase	binds plasminogen	21
Phosphoglycerate kinase	kinase	binds plasminogen	21
Phosphoglyceromutase	mutase	binds plasminogen	21
Transcription elongation factor	elongation factor	binds plasminogen	21
Thiol-specific antioxidant protein	antioxidant	binds plasminogen	21
glycerol 3-phosphate dehydrogenase	dehydrogenase	binds plasminogen	24
high-affinity glucose transporter 1	sugar transporter	complement inhibitor	25
*Enteamoeba histolytica*			
alcohol dehydrogenase (EhADH2)	dehydrogenase	Binds fibronectin, laminin, collagen	26
*Homo sapiens* (human)			
Hsp90α	chaperone	binds to bacterial pathogens	27

### 2.2. Eukaryotes

#### 2.2.1. Yeast

Eukaryotic gut microbiota also use intracellular proteins as cell surface adhesins. The yeast *Candida albicans* is a common part of the gut microbiome and an opportunistic pathogen that can cause candidiasis in immunocompromised individuals. It can also be found on biofilms on implanted medical devices. GAPDH is an enzyme in glycolysis and has many moonlighting functions in many species (Table 1). In *C. albicans*, it was found to bind plasminogen as well as fibronectin and laminin [21],[22]. *Candida* also uses several other proteins from glycolysis and gluconeogenesis to bind to plasminogen, enolase [23], fructose 1,6-bisphosphate aldolase [21], phosphoglycerate kinase [21], and phosphoglyceromutase [21]. Glycerol 3-phosphate dehydrogenase, which functions in glycerol accumulation, is also an adhesion [24]. Three proteins involved in protection from alcohol, hydrogen peroxide and antioxidants also bind to plasminogen: alcohol dehydrogenase (ADH1) [21] which protects cells from ethanol, peroxisomal catalase (CTA1) [21], which protects cells from the toxic effects of hydrogen peroxide, and a thiol-specific antioxidant protein [21]. The transcription elongation factor TEF1, which promotes the GTP-dependent binding of aminoacyl-tRNA to the A-site of the ribosome during protein biosynthesis, is also a cell surface plasminogen binding protein [21].

Another protein in *Candida albicans* has a second function that is involved in modulation of the host's immune system. The high-affinity glucose transporter 1 is a sugar transporter that is also an inhibitor of the host's complement system [25]. It binds to the complement regulators FH and C4BP and protects the yeast from actions of the host's complement cascade.

#### 2.2.2. Amoeba

*Entamoeba histolytica* is a parasitic amoeba that infects the large bowel. It is estimated to infect about 50 million people worldwide, usually asymptomatically, but it can sometimes enter the epithelial cell layer and result in a lethal infection. It kills more than 50,000 people each year. An intracellular enzyme, alcohol dehydrogenase (EhADH2), which has both alcohol dehydrogenase and acetaldehyde dehydrogenase activity, can be found on the cell surface where it binds proteins of the host's extracellular matrix (ECM), including fibronectin, laminin, and type II collagen [26].

#### 2.2.3. Human protein moonlighting as a receptor and interacting with bacteria

In some cases of gut microbiota interactions with humans, it is the human cell that displays the moonlighting protein. Hsp90 on mammalian cell surfaces is involved in sensing bacterial proteins and lipopolysaccharide (LPS) and can aid in initiating an immune response. The cell surface protein JlpA from *Campylobacter jejuni*, a common cause of food poisoning, interacts directly and specifically with cell surface-exposed Hsp90 on human epithelial cells [27]. Binding to Hsp90 results in the activation of proinflammatory immune responses through signaling pathways involving NF-κB and p38 MAP kinase. It's not clear how the signal crosses the cell membrane because Hsp90 does not contain a transmembrane domain, but there must be at least one additional cell surface protein interacting with Hsp90 that can transduce the signal into the cell.

## 3. Other intracellular proteins found on the cell surface

The moonlighting proteins described above and many other intracellular proteins have also been observed on the surface of these and other species through larger scale proteomics studies of cell surface proteins [31]. Proteomics studies of *E. coli* identified elongation factor Tu, D-tagatose 1,6-bisphosphate aldolase 2, and isocitrate lyase on the cell surface [32]. Proteins found on the surface of *Enterococcus faecalis* included elongation factors G and Tu, tyrosine—tRNA ligase, alanine—tRNA ligase, chaperone protein DnaK, phosphoglycerate mutase, pyruvate kinase, fructose 1,6-bisphosphate aldolase, enolase, GAPDH, formate acetyltransferase, and adenylate kinase [33]. Some of these proteins and many others were found on the surface of *Listeria monocytogenes*, including GroEL, DnaK , GAPDH, enolase, translation elongation factors tsf and G, pyruvate kinase, cysteine synthase, phosphoglycerate kinase, glutamate dehydrogenase, transketolase, branched chain amino acid aminotransferase, glucose 6-phosphate isomerase, and triosphosphate isomerase [34]. Ten proteins often found to moonlight were found on the surface of *Bifidobacterium animalis* ssp. *Lactis* KLDS 2.0603, including GroEL, GroES, EF-ts, GAPDH, transaldolase, DnaK, enolase, phosphoglucosamine mutase, bile salt hydrolase, and ribosomal protein L13, and many of the same proteins were found on the surface of *Lactobacillus acidophilus* NCFM [41]. Kinoshita and coworkers showed that phosphate buffer can wash over a dozen intracellular proteins from the surface of intestinal lactic acid bacteria, including GroEL, enolase, EF-Tu, phosphoglyceromutase, triosephosphate isomerase, DnaK, phosphofructokinase, and phosphoglycerate kinase, and at least some of these proteins bind to porcine intestinal mucosa [38].

The results of several proteomics studies show that changes in dietary components can cause changes in the expression or secretion of intracellular/surface moonlighting proteins and thereby might have an effect on the strength of interaction of some probiotics with host cells. Celebioglu and coworkers showed that several intracellular proteins are found on the surface of *Lactobacillus acidophilus* and that growth in the presence of different carbon sources, plant polyphenols, or ‘prebiotics’ (molecules like raffinose that humans cannot digest but intestinal bacteria can) affected the level of expression of several of these proteins on the cell surface, including EF-Tu and pyruvate kinase [39],[42],[43]. In addition, Montoro and coworkers showed that the levels of surface expression of phosphoglucomutase and several other proteins normally found in the cytoplasm varied in strains of the probiotic *Lactobacillus pentosus* that varied in their strength of adhesion to porcine mucin [44].

Overall, a wide variety of intracellular proteins have been found on the surface of gut microbiota. From the results of the proteomics studies, it was not determined if the proteins perform the same function on the cell surface as in the cell or if they perform a different function there, so additional experiments would be needed before determining if they are true moonlighting proteins. In some cases, observing a protein on the cell surface could be due to challenges in the experimental method, for example, proteins that are interacting firmly with a cytoplasmic domain of a transmembrane protein complex, might be misidentified as being on the cell surface. Further experiments will be needed to confirm if the intracellular proteins identified in the surface proteomics studies also function as adhesins.

## 4. Discussion

The observation of many intracellular proteins with a second function on the cell surface raises several questions.

First, the mechanisms by which these intracellular proteins are secreted while many other highly abundant intracellular proteins remain in the cell is not known. They do not contain signal sequences for secretion through the canonical Sec secretion pathway or other known motifs required for noncanonical secretion pathways. An analysis of one hundred intracellular/surface moonlighting proteins found that the intracellular/surface moonlighting proteins have physicochemical features that are similar to other cytosolic proteins [35].

It is also not known how most of the intracellular/surface moonlighting proteins bind to the cell surface. The secretion pathways and receptors or other mechanisms for binding to the cell surface might be versions of the known pathways and receptors or might involve novel processes. Because many of the intracellular/surface proteins described above are widely conserved in evolution and play important roles in human cells, their catalytic mechanisms might not be good targets in the development of novel therapeutics to treat infections. Instead, elucidating how these proteins are secreted and bound to the bacterial cell surface might lead to the identification of processes and proteins that could serve as targets for therapeutics.

Many of the intracellular proteins found to be adhesins on pathogens are homologues of proteins found to be adhesins on the surface of probiotic species, for example Hsp60. This might help explain why some probiotics appear to be able to compete with or crowd out pathogenic species. This use of homologous proteins by pathogens and probiotics also means that it would be important to find treatments that affect only those protein homologues in the pathogens and not in the probiotic species, or perhaps it would be possible to find a way to use these proteins to help probiotic species to displace pathogens and improve the balance of bacterial species in the gut.

It is also interesting that both pathogens and probiotics have cell surface proteins that bind to plasminogen. Plasminogen can be converted to plasmin, an active protease that can be used to degrade host extracellular matrix and basement membrane and thereby enable pathogens to invade nearby tissues [28]–[30], but it's not clear why probiotic species bind plasminogen.

## 5. Conclusions

Intracellular/surface moonlighting proteins are used by species described above that can cause serious intestinal infections. For example, *Clostridium difficile* can cause life-threatening intestinal inflammation and diarrhea and leads to tens of thousands of deaths in the US each year. An imbalance between pathogenic and probiotic species is also associated with inflammatory bowel disease (ulcerative colitis and Crohn's disease), chronic diseases that affect over 1 million people in the US alone and is increasing in prevalence worldwide [36],[37]. Understanding more about intracellular/surface moonlighting proteins, their mechanisms of secretion, their mechanisms for binding to the cell surface, their interactions with the host, and the competition with other species could be important for finding improved methods to prevent or treat many serious infections and chronic diseases of the gut for many people.
